# Synthetic Biology Facilitates Antimicrobials Discovery

**DOI:** 10.3390/antibiotics12030578

**Published:** 2023-03-15

**Authors:** Linquan Bai, Zixin Deng, Yaojun Tong

**Affiliations:** State Key Laboratory of Microbial Metabolism, Joint International Research Laboratory of Metabolic and Developmental Sciences, School of Life Sciences and Technology, Shanghai Jiao Tong University, Shanghai 200240, China

We are currently facing two big global challenges: antibiotics shortage and multidrug resistance. Since the “Golden Age” of natural products-based antibiotic discovery in the mid-20th century, we have only discovered a very limited number of new antibiotics. This indicates that the traditional antibiotic discovery pipeline is drying up. Moreover, the misuse and overuse of limited classes of antibiotics have caused the emergence and spread of multidrug resistance. Fortunately, the advance of synthetic biology brings us new opportunities for antibiotic discovery. A starting place could be the genomic (gene) and related enzymatic information with the “bottom-up” strategy [1], empowered by “cutting-edge” biotechnology such as CRISPR-based genome editing [2], to facilitate antibiotic discoveries from natural resources.

Several important studies [3,4,5,6,7] on synthetic biology-assisted antibiotic research have been published recently, and we strongly feel that it is about time to re-attract researchers’ attention to antibiotic discovery using cutting-edge concepts such as synthetic biology. This idea encourages us to organize this Special Issue on “Synthetic Biology Brings New Opportunity for Antibiotics Discovery”. This Special Issue covers a broad range of antibiotic research, comprising three review articles and seven original research papers.

In the review article written by Huang and colleagues [8], the biosynthesis and working mechanisms of specific plant-derived antimicrobial agents such as artemisinin, oleanolic acid, berberine, colchicine, and baicalin are summarized and discussed with a goal to provide insights for the future discovery and development of such agents.

The review by Wang and colleagues [9] summarizes the diverse secondary metabolites that are produced by a popular edible and medicinal mushroom, *Inonotus hispidus.* Its secondary metabolites are mainly polyphenols and triterpenoids, with multiple bioactivities, including anticancer, immunomodulatory, anti-inflammatory, antioxidant, antimicrobial, and enzyme inhibitory activities. *I. hispidus* is a promising source of bioactive compounds, including antibiotics for health promotion and functional food development.

To address the challenge of the shortage of antifungals, Zhong and colleagues have written a review [10] that focuses on how to use synthetic biology to facilitate the development of novel antifungal drugs that originate from traditional Chinese medicine derived from natural products.

In the study conducted by Geng and colleagues, the mechanisms of antibiotic resistance and genes associated with antibiotic resistance in *Zymomonas mobilis* are deeply investigated using bioinformatics and CRISPR/Cas12a-based genome-editing technology. Six ampicillin-resistant genes are identified, and their related mutants are constructed. It shows that ZMO0103 is the key to ampicillin resistance in *Z. mobilis* and how other ampicillin-resistant genes may have a synergistic effect [11]. This study could lay the foundation for further studies of other antibiotic resistance mechanisms.

The research article conducted by Yang and colleagues reports a novel global regulatory protein, SspH, which is widespread in *Streptomyces*, and this was found to play an important role in controlling different types of antibiotic production. This study provides insight into the regulation of antibiotic biosynthesis and the potential targets for future antibiotic discovery and production [12].

Liu and colleagues, in their research paper, aimed to identify the genes that are involved in the export of the anti-coccidiosis agent salinomycin produced by *Streptomyces albus* BK3-25. By analyzing transcriptomes, eight putative transporter genes were found and proven to positively impact salinomycin exports. The overexpression of these genes will lead to an increase in the titer of salinomycin and improved self-resistance. The study provides a transcriptome-based strategy for improving the titer of salinomycin and identifying transporter genes for other antibiotics [13].

In their research article, Xue and colleagues found that L-kynurenine (Kyn) is a building block for the biosynthesis of bioactive natural products. They used a genome-mining approach and discovered a biosynthetic gene cluster (BGC) from *Neosartorya pseudofischeri* that can produce pseudofisnins: novel 1-benzazepine-containing compounds. A methyltransferase named PseC was identified as a crucial enzyme in this BGC, catalyzing di-methylation in an amine group [14].

Using a combinatory strategy of genome-mining and OSMAC (one strain of many compounds), Zhang and colleagues identified two new and seven known cyclodipeptide derivatives from *Streptomyces* sp. 26D9-414. The new compound 2 showed similar cytotoxicity to cisplatin in treating various cancer cells [15], which is promising for further investigations.

In the study conducted by Al-Thubaiti and colleagues, metal cefotaxime complexes of Ca(II), Cr(III), Zn(II), Cu(II), and Se(VI) were synthesized and characterized. They then investigated the effects of cefotaxime and cefotaxime metal complexes on oxidative stress and their activity against cancer cells (HepG-2) and bacteria (*Bacillus subtilis* and *Escherichia coli*). The results showed that cefotaxime metal complexes with Zn and Se had high antioxidant activity against HepG-2 cells, and they also presented potent antibacterial activities at low concentrations [16].

El-Megharbel and colleagues, in their research article, investigated the formation of Mg(II), Fe(III), Cu(II), Zn(II), and Se(IV) complexes of the antibiotic ceftriaxone (CFX). Furthermore, they also investigated the effect of CFX and its metal complexes on oxidative stress and tissue injury in rats. It showed that CFX and its metal complexes, especially CFX/Zn, had high antibacterial activity [17].

I hope that readers find the articles in this Special Issue interesting and inspiring for their own research. Joining together, let us bring society another “Golden Age” of antibiotic discovery!
